# Acyclic Cucurbit[n]uril-Enabled Detection of Aflatoxin B1 via Host–Guest Chemistry and Bioluminescent Immunoassay

**DOI:** 10.3390/toxins17030104

**Published:** 2025-02-25

**Authors:** Shaowen Wu, Ke Feng, Jinlu Niu, Jintao Xu, Hualian Mo, Xiaoman She, Shang-Bo Yu, Zhan-Ting Li, Shijuan Yan

**Affiliations:** 1 State Key Laboratory of Swine and Poultry Breeding Industry, Agro-Biological Gene Research Center, Guangdong Academy of Agricultural Sciences, Guangzhou 510640, China; wushaowen@agrogene.ac.cn (S.W.); niujinlu2021@163.com (J.N.); xujintao_1999@163.com (J.X.); mhl981585@163.com (H.M.); 2 State Key Laboratory of Organometallic Chemistry, Shanghai Institute of Organic Chemistry, Chinese Academy of Sciences, University of Chinese Academy of Sciences, 345 Lingling Lu, Shanghai 200032, China; fengke@sioc.ac.cn (K.F.); ztli@mail.sioc.ac.cn (Z.-T.L.); 3 Guangdong Provincial Key Laboratory of High Technology for Plant Protection, Institute of Plant Protection, Guangdong Academy of Agricultural Sciences, Guangzhou 510640, China; shexiaoman@gdppri.com

**Keywords:** aflatoxin B1, host–guest chemistry, acyclic cucurbit[n]urils, bioluminescent immunoassay, supramolecular recognition

## Abstract

Aflatoxin B1 (AFB1), a highly toxic secondary metabolite produced by Aspergillus species, represents a significant health hazard due to its widespread contamination of agricultural products. The urgent need for sensitive and sustainable detection methods has driven the development of diverse analytical approaches, most of which heavily rely on organic solvents, posing environmental challenges for routine food safety analysis. Here, we introduce a supramolecular platform leveraging acyclic cucurbit[n]uril (acCB) as a host molecule for environmentally sustainable AFB1 detection. Screening various acCB derivatives identified acCB6 as a superior host capable of forming a stable 1:1 complex with AFB1 in an aqueous solution, exhibiting a high binding affinity. Proton nuclear magnetic resonance (1H NMR) spectroscopy confirmed that AFB1 was deeply encapsulated within the host cavity, with isothermal titration calorimetry (ITC) experiments and molecular dynamics simulations further substantiating the stability of the interaction, driven by enthalpic and entropic contributions. This supramolecular host was incorporated into a scaffold-assembly-based bioluminescent enzyme immunoassay (SA-BLEIA), providing a green detection platform that rivals the performance of traditional organic solvent-based assays. Our findings highlight the potential of supramolecular chemistry as a foundation for eco-friendly mycotoxin detection and offer valuable insights into designing environmentally sustainable analytical methods.

## 1. Introduction

Aflatoxins, particularly aflatoxin B1 (AFB1), are among the most toxic secondary metabolites produced by *Aspergillus* species, frequently contaminating staple agricultural commodities such as peanuts, corn, cereals, and their derivatives [1]. AFB1 has been classified as a Group 1 carcinogen by the International Agency for Research on Cancer due to its potent carcinogenic, mutagenic, and immunosuppressive effects, even at trace levels [1,2]. These serious health risks, combined with the widespread occurrence of AFB1, have prompted stringent regulatory limits on AFB1 concentrations in food and feed products, typically ranging from 2 to 20 μg/kg [3,4]. Consequently, developing sensitive, reliable, and scalable methods for AFB1 monitoring remains a critical priority.

Current AFB1 detection techniques predominantly rely on chromatographic methods, including high-performance liquid chromatography (HPLC) and liquid chromatography-tandem mass spectrometry (LC-MS), alongside immunological assays such as enzyme-linked immunosorbent assay (ELISA) [5,6,7]. While HPLC and LC-MS deliver excellent sensitivity and specificity, their complex instrumentation, high operational costs, and reliance on skilled technicians limit their scalability for routine applications. Immunoassays, on the other hand, are better suited for high-throughput analysis [8,9]. However, a shared limitation across these methodologies is their heavy dependence on organic solvents for sample preparation and standard solutions, which raises concerns about their environmental sustainability. Addressing these challenges requires the development of green alternatives that can balance sensitivity, reliability, and environmental impact.

Host–guest chemistry has emerged as a powerful tool for molecular recognition, enabling the sequestration and detection of toxins, drugs, and other contaminants [10,11,12,13,14,15,16,17,18]. One notable example is the inclusion–antagonism mechanism of sugammadex, which efficiently removes residual neuromuscular blocking agents in clinical applications. Supramolecular hosts such as cyclodextrins, calixarenes, cucurbiturils, and pillararenes exhibit excellent biocompatibility and form strong complexes with target molecules, making them suitable for in vivo and in vitro applications. Among these, acyclic cucurbit[n]urils (acCBs) are particularly promising due to their high water solubility and adaptable cavities, which can flexibly bind guests of varying sizes. Recent advancements have shown that modifying acCBs with exocyclic substituents can fine-tune their backbone curvature and steric properties, enhancing their binding versatility [16,19]. Such functional adaptability makes acCBs ideal candidates for various guest-binding applications, including toxin detection [20,21].

**Figure 1:**
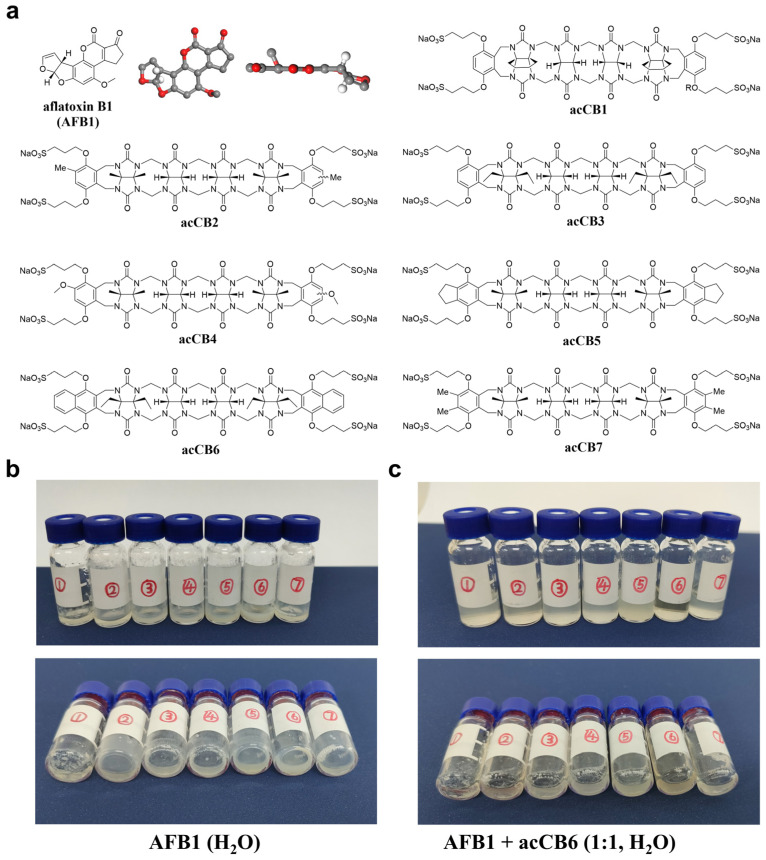
Chemical structures of acyclic cucurbit[n]urils and their ability to enhance the solubility of aflatoxin B1 (AFB1). (**a**) Chemical structure of AFB1 alongside the structures of acCB derivatives, illustrating their substituents and cavity dimensions. (**b**,**c**) Visual representation of AFB1 dissolution enhancement in aqueous solution. Sample vials containing AFB1 with various acCBs (vials 1–7 corresponding to acCB 1–7) at 0 molar equivalents (**b**) and 1.0 molar equivalents (**c**). Top and side views of the vials are provided.

AFB1 possesses a difuranocoumarin-like structure characterized by a highly planar conjugated aromatic system (Figure 1a). The aromatic moieties of acCBs provide opportunities for π-π stacking interactions with AFB1, potentially forming tightly packed complexes. Building on the binding versatility of acCBs, this study investigates their application in AFB1 detection. Spectroscopic, thermodynamic, and computational analyses revealed that acCB6 forms a highly stable 1:1 complex with AFB1 in aqueous solution. Furthermore, integrating this host–guest system into a scaffold-assembly-based bioluminescent enzyme immunoassay (SA-BLEIA) enabled sensitive, environmentally sustainable AFB1 detection. This work not only establishes the utility of acCBs for mycotoxin detection but also underscores their potential for advancing green analytical methodologies through host–guest chemistry.

## 2. Results

### 2.1. Dissolution Enhancement of AFB1 by acCBs

To evaluate the potential of acCBs as solubilizing agents for AFB1, we systematically screened a series of acCB derivatives with varying structure features (Figure 1a). While acCBs have previously demonstrated efficacy in enhancing the solubility of poorly soluble pharmaceuticals, their ability to dissolve AFB1 in water—given its planar spatial structure and extremely low solubility (10–30 μg/mL)—remains uncertain [22]. Initial solubility experiments were performed via visual inspection. AFB1 was dissolved in water in sample vials, followed by the addition of acCBs at 1.0 molar equivalents (Figure 1b,c). Among the derivatives tested, acCB6 exhibited exceptional dissolution efficacy, forming a completely transparent solution, indicative of full AFB1 either remaining as visible deposits on the vial walls or suspended in the solution. These observations suggest that the naphthalene substituents of acCB6 provide an optimal cavity size and aromatic surface for effective AFB1 encapsulation. This preliminary screening identified acCB6 as the most effective solubilizing agent for AFB1.

### 2.2. Spectroscopic Evidence for 1:1 Host–Guest Complex Formation

**Figure 2:**
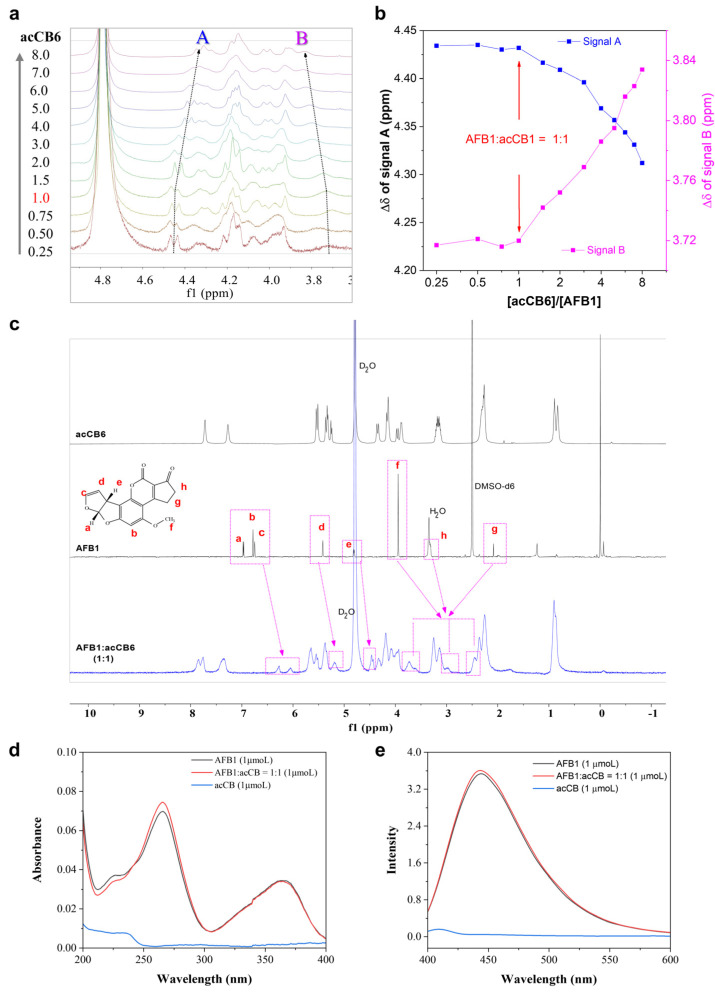
NMR, UV-vis, and fluorescence spectroscopic characterization of the acCB6-AFB1 complex. (**a**) ^1^H NMR titration spectra showing the evolution of chemical shift changes in the 3.6–5.0 ppm region upon the stepwise addition of acCB6 (0.8–25.6 mM, 0.25–8.0 equivalents) to AFB1 (3.2 mM). (**b**) Signal shift analysis for A and B as a function of [acCB6]/[AFB1] ratio, highlighting a 1:1 binding stoichiometry. (**c**) Comparative ^1^H NMR spectra of free acCB6 (3.2 mM, D_2_O), free AFB1 (3.2 mM, DMSO-d6), and their 1:1 mixture (3.2 mM, D_2_O) at 25 °C, with key proton assignments of AFB1 (a–g) indicated. (**d**) UV–visible absorption spectra and (**e**) fluorescence emission spectra of acCB6 (1 μM), AFB1 (1 μM), and their 1:1 complex (1 μM) in H_2_O at 25 °C, demonstrating spectral changes upon complex formation.

To elucidate the molecular interactions underpinning acCB6’s superior solubilizing capability, we performed spectroscopic analyses. NMR titration experiments provided detailed insights into the binding mechanism and stoichiometry of the acCB6-AFB1 interaction. Stepwise addition of acCB6 (0.25–8.0 equivalents) to AFB1 led to systematic chemical shift changes in the diagnostic proton signals within the ^1^H NMR spectra (Figure 2a). Significant upfield shifts were observed for signals A and B, indicative of AFB1 inclusion within the acCB6 cavity. Binding stoichiometry analysis revealed an inflection point at a 1:1 molar ratio (Figure 2b), supporting the formation of a well-defined 1:1 host–guest complex. Given AFB1’s extremely low solubility in water, these NMR observations further support the fact that acCB6 successfully solubilized AFB1.

Comparative ^1^H NMR spectra of free acCB6, free AFB1, and their equimolar mixture further confirmed this interaction. Notable upfield shifts in AFB’s H-a, H-b, H-c, and H-f proton signals (Figure 2c), suggest deep encapsulation of nearly the entire AFB1 molecule with acCB6 cavity. Complementary UV–visible and fluorescence spectroscopy analyses revealed slight increases in AFB1’s absorbance and fluorescence upon complexation with acCB6 (Figure 2d,e), corroborating the host–guest complex formation. These spectroscopic findings establish acCB6 as a highly effective host molecule for AFB1 encapsulation in aqueous media.

### 2.3. Thermodynamic Analysis and Molecular Dynamics Simulations Reveal the Driving Forces of Complex Formation

**Figure 3:**
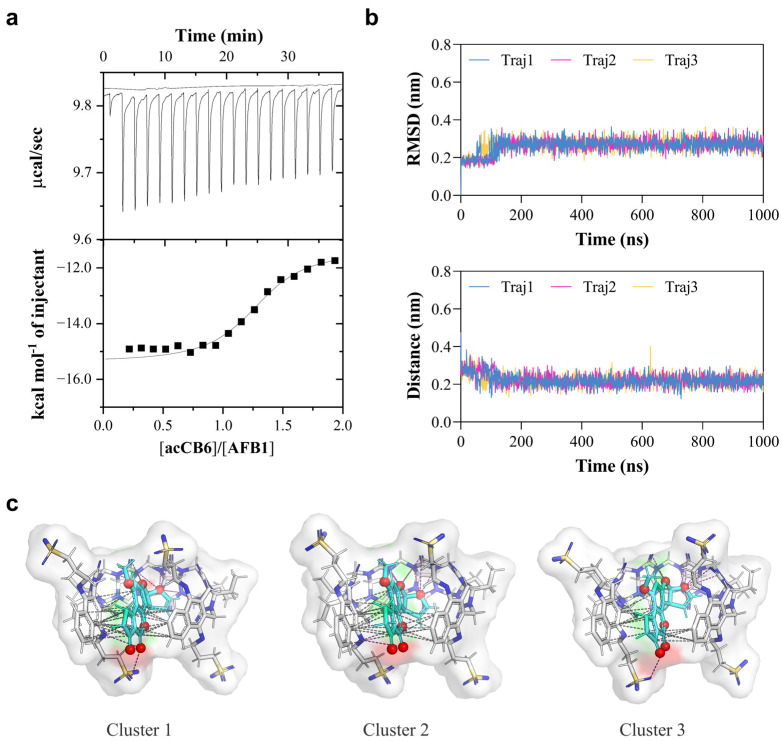
ITC and molecular dynamics analyses of acCB6-AFB1 interactions. (**a**) ITC titration curve showing raw calorimetric data and integrated heat changes for the addition of acCB6 (0.1 mM) to AFB1 (0.01 mM) at 25 °C. The solid line represents the best fit to a 1:1 binding model. (**b**) Molecular dynamics simulation results, including an RMSD plot of the acCB6-AFB1 complex (top) and the center-of-mass distance between acCB6 and AFB1 (bottom) over 1000 ns across three independent trajectories. (**c**) Representative structures from cluster analysis of MD trajectories showing three dominant binding modes (Clusters 1–3). AFB1 is depicted in cyan, acCB6 in gray, with polar interactions (magenta) and π-π stacking interactions (gray) shown as dashed lines. Polar O/N atoms are colored red in AFB1 and blue in acCB6. The host cavity surface is rendered in transparent white to illustrate the encapsulation of AFB1.

To quantitatively investigate the driving forces of the acCB6-AFB1 interaction, we conducted isothermal titration calorimetry (ITC) experiments. Titration of acCB6 (0.1 mM) into AFB1 solution (0.01 mM) at 25 °C produced well-defined binding isotherms that fit a 1:1 binding model (Figure 3a). The binding constant (Ka) was determined to be (3.02 ± 4.7) × 10^6^ M^−1^, corresponding to a favorable Gibbs free energy change (ΔG) of −8.84 kcal/mol (Table 1). The interaction was driven by both enthalpic contributions (ΔH = −3.96 ± 0.582 kcal/mol) and entropic gains (−TΔS = −4.88 kcal/mol), highlighting the thermodynamic stability of the complex. The stoichiometry value (N = 1.27 ± 0.08) further confirmed the 1:1 binding mode observed in NMR studies.

To gain atomic-level insights into the binding mechanism of the acCB6-AFB1 complex, molecular dynamics (MD) simulations were performed. Three independent 1000 ns trajectories were analyzed to assess stability and binding modes (Figure 3b). Root-mean-square deviation (RMSD) analysis indicated rapid equilibration and stable complex formation, with fluctuations stabilizing at approximately 0.3 nm. The center-of-mass distance between acCB6 and AFB1 remained consistently close (~0.2 nm), confirming a stable host–guest association throughout the simulations. Energy component analysis revealed a total binding energy of −76,488.2 kJ/mol, consisting of potential energy (−92,910.53333 kJ/mol), Coulombic short-range interactions (−106,370.0 kJ/mol), and Lennard-Jones interactions (13,364.53333 kJ/mol). The large negative Coulomb energy highlights strong electrostatic interactions between the host and guest molecules, while the positive Lennard-Jones term reflects the balance between attractive van der Waals forces and repulsive steric interactions at close-contact distances (Table 2). These results align with the favorable energetics observed in ITC measurements and MD calculations, underscoring the structural stability of the complex.

Cluster analysis of the MD trajectories was conducted to identify predominant binding modes. Representative structures from the top three clusters showed the AFB1 molecule consistently embedded deeply within the acCB6 cavity. In all clusters, the complex was stabilized by an intricate network of intermolecular interactions, visualized as polar contacts (magenta dashed lines, Figure 3c) and π-π stacking interactions (gray dashed lines). These interactions contribute directly to the observed Coulomb and Lennard-Jones energies and provide a molecular explanation for the favorable enthalpic contributions measured by ITC. The recurrent presence of these interactions across binding modes supports the specificity and strength of the acCB6-AFB1 recognition mechanism.

### 2.4. Development of acCB6-Enabled Aqueous Detection Assay for AFB1

**Figure 4:**
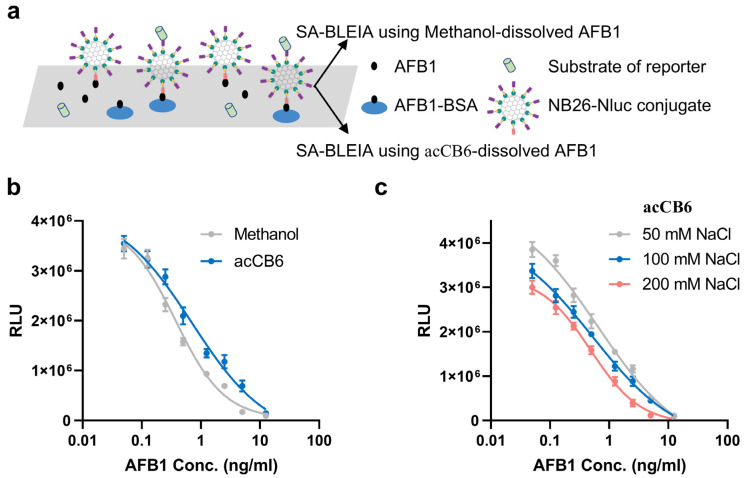
Performance of the scaffold-assembly-based bioluminescent enzyme immunoassay (SA-BLEIA) analysis with AFB1 standards dissolved in different solutions. (**a**) Schematic representation of the SA-BLEIA detection principle, comparing traditional methanol-dissolved AFB1 standards with acCB6-dissolved AFB1 standards. (**b**) Standard curves illustrating the detection performance of methanol-dissolved AFB1 (gray) and acCB6-dissolved AFB1 (blue) standards, highlighting differences in sensitivity. (**c**) Influence of ionic strength on SA-BLEIA performance using acCB6-dissolved AFB1 standards at varying NaCl concentrations (50, 100, and 200 mM).

With acCB6 established as a potent host for AFB1 solubilization, its potential as a green alternative to organic solvents in analytical detection was explored. Specifically, we investigated its application in a previously developed scaffold-assembly-based bioluminescent enzyme immunoassay (SA-BLEIA), comparing traditional methanol-dissolved AFB1 standards with the novel acCB6-dissolved AFB1 standards (Figure 4a) [9]. In this assay, the AFB1 present in the solution engages in a competitive binding interaction with hapten AFB1-BSA that is immobilized on the microplate surface, vying for attachment to nanobody–luciferase conjugates. As the concentration of AFB1 escalates, the bioluminescence signal, measured in Relative Light Units (RLUs), correspondingly diminishes. Initial experiments demonstrated that while the acCB6-based method produced detection curves comparable to the conventional approach, it exhibited reduced sensitivity. The IC_50_ value increased from 0.3730 ng/mL (methanol) to 0.7045 ng/mL (acCB6) (Figure 4b, Table 3). Given the MD simulations showing strong electrostatic interactions in the acCB6-AFB1 complex, we hypothesized that modulating ionic strength could improve assay performance.

To evaluate the effect of ionic strength, the SA-BLEIA system was tested under varying NaCl concentrations ranging from 50 to 200 mM. The results revealed a clear trend of enhanced assay sensitivity with increasing salt concentration (Figure 4c, Table 3). The IC_50_ values improved progressively, decreasing from 0.6460 ng/mL at 50 mM NaCl to 0.5229 ng/mL at 100 mM NaCl, and further to 0.4825 ng/mL at 200 mM NaCl. Notably, the bioluminescence intensity displayed a concentration-dependent response to ionic strength, with higher NaCl concentrations slightly reducing signal intensity across the AFB1 concentration range. Despite this reduction, the improved IC50 values indicate enhanced AFB1 detection sensitivity under higher salt conditions.

The observed improvement of sensitivity with increasing ionic strength is likely due to the modulation of electrostatic interactions between the acCB6-AFB1 complex and assay components. Under low-salt conditions, strong electrostatic forces may limit AFB1 availability, whereas increased ionic strength optimizes the balance between complex stability and AFB1 accessibility for immunodetection. At 200 mM NaCl, the detection sensitivity approached that of the traditional methanol-based method, with IC50 values of 0.4825 ng/mL and 0.3730 ng/mL, respectively. These findings highlight acCB6 as a promising green alternative for AFB1 detection, particularly when paired with optimized salt conditions.

## 3. Discussion

This study demonstrates the successful development of a highly effective host–guest system for AFB1 detection using acCBs. Through screening seven acCB derivatives, we identified acCB6 as the optimal host molecule, forming a stable 1:1 complex with AFB1 in an aqueous solution and exhibiting a remarkable binding affinity (Ka = 3.02 × 10^6^ M^−1^); the high binding strength arises from a synergistic combination of enthalpic and entropic contributions. By integrating this host–guest system into an SA-BLEIA, we observed salt-dependent assay performance, highlighting the practical versatility of acCB6. Unlike traditional AFB1 detection methods, which typically rely on organic solvents, our approach emphasizes the feasibility of aqueous-phase detection, offering an environmentally sustainable alternative. Notably, the assay’s sensitivity improved with increasing ionic strength, as reflected in the IC_50_ values, underscoring the delicate balance between host–guest binding affinity and AFB1 accessibility for immunodetection, both of which are modulated by ionic conditions.

The superior performance of acCB6 can be attributed to well-established principles of host–guest chemistry and molecular recognition [10,23]. The naphthalene moieties in acCB6 provide extensive aromatic surfaces, facilitating efficient π-π stacking interactions with the conjugated system of AFB1, which are critical for the stabilization of the complex. Additionally, the exocyclic ethyl groups likely enhance encapsulation by endowing the cavity with structural adaptability [24,25]. These structural features align with prior studies, which have shown that acCBs with appropriately sized cavities and aromatic walls exhibit enhanced guest binding [20,26]. MD simulations further elucidate the critical role of electrostatic interactions and solvation effects in the host–guest binding process [10,27]. The simulations suggest that the salt-dependent sensitivity enhancement arises from ionic strength modulating these electrostatic interactions, offering valuable insights into the behavior of the system in aqueous solutions [28].

Our findings significantly advance the field of AFB1 detection by demonstrating that a cucurbituril-based system can achieve comparable sensitivity to traditional immunoassays without relying on organic solvents. This marks a significant departure from conventional methods that depend on organic solvents for the preparation of standard curves [9,29]. The acCB6-based system enables detection in aqueous conditions while maintaining high sensitivity, eliminating environmental risks associated with solvent use. This innovation enhances the practicality of AFB1 detection and promotes environmental sustainability, aligning with the global push toward reducing chemical waste in scientific and industrial applications. Additionally, our results build upon existing research on cucurbituril-based recognition systems.

Previous studies highlight the potential of acCBs to improve pharmaceutical solubility, although they have reported lower binding constants (Ka ~ 10^4^–10^5^ M^−1^) [21,30]. The exceptionally high binding affinity observed in this study represents a substantial improvement addressing the stringent regulatory requirements for AFB1 detection in food products. Integration of acCB6 with the SA-BLEIA platform offers notable advantages over existing detection systems. Beyond eliminating the need for organic solvents, our system maintains a high sensitivity level, making it suitable for diverse applications. This is particularly relevant in the context of recent advances in nanobody-based detection systems, which have similarly reduced organic solvent dependency in immunoassays. Our work complements this growing body of research, presenting an environmentally sustainable solution for mycotoxin detection [31].

However, some limitations of the current system warrant consideration. While increasing ionic strength enhances sensitivity, a reduction in absolute signal intensity is observed at higher salt concentrations. This may result from the competitive effects of ions on the binding process. Although the reduction does not significantly impact overall sensitivity, it highlights an area for further optimization. Future studies could focus on refining the structure of acCB6 to preserve high binding affinity while minimizing its sensitivity to ionic strength variations [23,32]. Overall, our findings deepen the understanding of host–guest chemistry in aqueous media and introduce a novel, eco-friendly approach for AFB1 detection. This system holds significant potential as a reliable tool for ensuring food safety, complementing traditional techniques like HPLC and LC-MS within regulatory frameworks. It offers a more rapid and cost-effective initial screening option for detecting aflatoxins and provides valuable references for detecting other harmful compounds in diverse food products.

## 4. Materials and Methods

### 4.1. Synthesis of acCBs

The synthesis of acCB1-7 was conducted following previously reported methods [18,19].

### 4.2. AFB1 Dissolution Promotion Experiments

To prepare AFB1-containing samples, a standard acetone solution of AFB1 (1 mg/mL) was used. A volume of 100 μL was deposited in clean glass vials, and the acetone was removed under vacuum in a drying oven to leave 0.1 mg of AFB1 adhered to the vial walls. Stock solutions of acCBs were prepared in deionized water and then sterilized using 0.22 μm filters. During dissolution experiments, acCB solutions were added to AFB1-coated vials in molar equivalent amounts (1.0 equivalents). Samples were sonicated for 5 min, and the degree of dissolution was visually evaluated.

### 4.3. NMR Experiments

NMR titration experiments were performed using a Bruker 500 MHz spectrometer (Bruker BioSpin GmbH, Rheinstetten, Germany). A stock solution of acCB6 in D_2_O (80 mM) was titrated in incremental volumes into NMR tubes containing 0.5 mg (1.6 × 10^−3^ mM) of AFB1, covering a molar ratio range of 0.25 to 8.0 equivalents. The residual non-deuterated solvents (δ 4.79 for D_2_O and δ 2.50 for DMSO-*d*_6_) were used as internal references for ^1^H NMR spectra.

### 4.4. UV–Visible Absorption Spectra

Stock solutions of acCB6 (1 mM) and AFB1 (1 × 10^−2^ mM) were prepared in ultrapure water and subsequently diluted to a final concentration of 1 μM. An aqueous solution of the AFB1-acCB6 complex (1 μM) was prepared by combining the diluted stock solutions. The UV–visible absorption measurements were performed using a Unico UV-4802 double-beam spectrophotometer (UNICO, Shanghai, China).

### 4.5. Fluorescence Emission Spectra

Fluorescence emission spectra were measured using a PerkinElmer FL6500 fluorescence spectrophotometer (PerkinElmer Inc., Waltham, MA, USA). The sample preparation followed the procedure outlined in Section 4.4. After 1 h of equilibration at room temperature, spectra were recorded using an excitation wavelength of 360 nm with a slit width of 20 nm. Emission spectra were measured at 440 nm with a slit width of 10 nm.

### 4.6. Isothermal Titration Calorimetry (ITC) Experiments

ITC experiments were conducted at 25 °C using a MicroCal PEAQ-ITC calorimeter (Malvern Panalytical, Malvern, UK). A solution of acCB6 (0.1 mM) was titrated into an AFB1 solution (0.01 mM) prepared in ultrapure water. Data were analyzed using the manufacturer’s software (MicroCal PEAQ-ITC Analysis Software 1.1.0.1262), employing a one-site binding model.

### 4.7. SA-BLEIA Procedure

The SA-BLEIA experiments were performed following previously described protocol [9]. High-binding white 96-well microplates were coated overnight at 4 °C with 100 μL of 5 μg/mL AFB1-BSA in PBS buffer (10 mM phosphate, pH 7.4). After incubation, the plates were blocked using 3% skim milk in PBS. Subsequently, 50 μL of 0.5 μg/mL nanobody–luciferase conjugates were pre-incubated with 50 μL of varying AFB1 (TMRM, Changzhou, China, 99% purity) concentrations prepared in PBS buffer with either 20% methanol (Sinopharm Chemical Reagent Co., Ltd., Shanghai, China) or 1.0 molar equivalents of acCB6. To evaluate salt-dependent performance, NaCl was added to the PBS buffer at final concentrations of 50, 100, or 200 mM. This mixture was added to the antigen-coated wells and incubated for 30 min at 37 °C. Plates were washed three times with PBST buffer (PBS containing 0.05% Tween-20), followed by the addition of luminescence substrates in the optimized assay buffer. Luminescence signals were recorded using a Varioskan Flash spectral scanning multimode reader (Thermo Fisher Scientific, Waltham, MA, USA). Standard curves were generated by plotting luminescence against the logarithm of AFB1 concentration and analyzed using four-parameter nonlinear regression for data analysis.

### 4.8. Molecular Dynamics (MD) Simulations

MD simulations were conducted using GROMACS 2020 with the GAFF force field for acCB6 and AFB1, and the modified TIP3P water model. A 150 mM NaCl concentration was included to neutralize the system [33,34]. Energy minimization was performed using the steepest descent algorithm for 50,000 steps. Equilibration was achieved through 1 ns simulations under the isothermal–isochoric (NVT) and isothermal–isobaric (NPT) ensembles [35], at 298 K, using the v-rescale thermostat and the Parrinello–Rahman barostat with a compressibility of 4.5 × 10^−5^ bar and a τp of 2 ps [36,37]. Production simulations were conducted for three independent 1000 ns runs with a 2 fs time step [38]. H bonds were constrained using the LINCS algorithm. The cutoff distance for electrostatic and van der Waals interactions was set to 1.2 nm. Long-range electrostatics were calculated using the particle mesh Ewald (PME) method [39]. Simulation trajectories were analyzed using GROMACS tools.

## Figures and Tables

**Table 1:** Thermodynamic parameters for acCB6 and AFB1 binding at 25 °C measured by ITC.

Syringe	Cell	N	Ka (M^−1^)	ΔH (kcal/mol)	−TΔS (kcal/mol)	ΔG (kcal/mol)
0.1 mM acCB6	0.01 mM AFB1	1.27 ± 0.08	(3.02 ± 4.7) × 10^6^	−3.96 ± 0.582	−4.88	−8.84

**Table 2:** Energy components of the acCB6-AFB1 complex from molecular dynamics simulations.

Total Energy (kJ/mol)	Potential (kJ/mol)	Coulomb (SR) (kJ/mol)	LJ (SR) (kJ/mol)
−76,488.2	−92,910.53333	−10,6370.0	13,364.53333

**Table 3:** Analytical performance comparison of SA-BLEIA with methanol and acCB6 as solvents for AFB1 standards under varied buffer conditions.

Buffer Conditions	IC_50_ (ng/mL)	R^2^
20% methanol	0.3730	0.9837
acCB6	0.7045	0.9814
acCB6 + 50 mM NaCl	0.6460	0.9885
acCB6 + 100 mM NaCl	0.5229	0.9926
acCB6 + 200 mM NaCl	0.4825	0.9924

## Data Availability

The original contributions presented in this study are included in the article. Further inquiries can be directed to the corresponding author.
